# Recommendations for the use of pediatric data in artificial intelligence and machine learning ACCEPT-AI

**DOI:** 10.1038/s41746-023-00898-5

**Published:** 2023-09-06

**Authors:** V. Muralidharan, A. Burgart, R. Daneshjou, S. Rose

**Affiliations:** 1 Department of Dermatology, Stanford University, Stanford, USA; 2 Department of Anesthesiology, Perioperative, and Pain Medicine, Stanford University, Stanford, USA; 3 Department of Biomedical Data Science, Stanford University, Stanford, USA; 4 Department of Health Policy, Stanford University, Stanford, USA

**Keywords:** Health policy, Paediatric research

## Abstract

ACCEPT-AI is a framework of recommendations for the safe inclusion of pediatric data in artificial intelligence and machine learning (AI/ML) research. It has been built on fundamental ethical principles of pediatric and AI research and incorporates age, consent, assent, communication, equity, protection of data, and technological considerations. ACCEPT-AI has been designed to guide researchers, clinicians, regulators, and policymakers and can be utilized as an independent tool, or adjunctively to existing AI/ML guidelines.

## The interests of children must be protected in artificial intelligence research

While a number of medical devices have been formally licensed for usage in children, there remains to date no guidance on the ethical use of pediatric data in artificial intelligence or machine learning (AI/ML) research^1^. To ensure fundamental ethical principles are prioritized through the ideation, development, iteration, deployment, and evaluation of AI/ML studies, researchers have highlighted the importance of formal ethics review and reporting procedures to improve safety and promote equity, and these efforts must be inclusive of the pediatric community^2,3^.

One definition of “algorithmic bias” refers to systematic inaccuracies in an AI/ML algorithm, causing potentially erroneous outputs that can result in the misclassification of select patients or subgroups and lead to actual harm^4^. Children and young people (CYP) under the age of eighteen are underrepresented in research, with pediatric studies presenting age-specific challenges that span ethical, legislative, financial, and relational domains^5^. Further, concerns about racial and gender disparities in pediatric research have been expressed, with calls to improve demographic reporting. It is important these considerations are accounted for in the development of pediatric AI/ML technology^6–9^.

If best practice standards are not established for CYP, the rapid expansion of AI/ML research has the potential to widen existing gaps. It is, therefore, crucial to define age-specific safety measures to prevent algorithmic bias and attain equitable, ethical, and appropriate representation of CYP.

### Age as a source of algorithmic bias

Sources of algorithmic bias that affect CYP may arise from a lack of transparency in participant age reporting, a lack of clinically and developmentally appropriate representation of children, and inappropriate generalizations made to the pediatric population from adult data, and vice versa. We describe the effect of age as a source of algorithmic bias using the term “age-related algorithmic bias”.

**Fig. 1:**
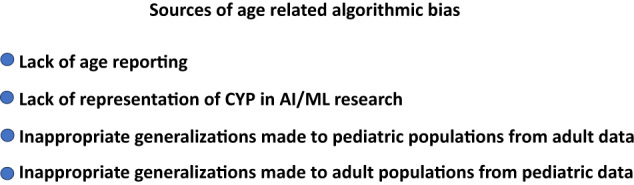
Sources of age-related algorithmic bias.

ACCEPT-AI is a framework of ethical principles and key recommendations for pediatric data utilization and the assessment of age-related algorithmic bias in AI/ML research for researchers, regulators, policymakers, and clinicians. ACCEPT-AI has been designed for independent usage to uphold ethical standards, and/or for adjunctive integration into existing guidelines such as CONSORT-AI^10^ and SPIRIT-AI^11^, and emerging guidelines such as TRIPOD-AI, PROBAST-AI^12^, STARD-AI^13^, and QUADAS-AI^14^ as well as future guidelines (Fig. 1).

### Pediatric data has age-specific considerations and should be viewed distinctly from adults

Pediatric health is heterogeneous, encompassing a range of developmental stages from birth to adolescence. Disease incidence, prevalence, presentation, outcome, and prognosis vary significantly between adult and pediatric populations, with vast physiologic and anatomic differences. Clinical needs, assessments, and treatment approaches are therefore distinct in both populations^15^.

Pediatric data presents unique ethical and practical challenges in conducting research. Such research must be grounded in ethical principles of autonomy and respect for persons, beneficence, non-maleficence, and justice^16^. Approaches to study enrollment, conduct, and implementation differ significantly from adult patients, with consenting, parental or guardian roles, and data protection necessitating tailored considerations for safe and age-appropriate care^5^. The U.S. Department of Health and Human Services requires special protections for CYP involved in research, defining standards that are more stringent than those of adults^17^. Researchers have emphasized the importance of including protected groups in research such that they are represented equitably^5^.

Typical developmental stages in the pediatric population present complexities and inherent differences in approach to study conception, design, data collection, usage, application, interpretability, and translation of research^18^. This variation is particularly important in the context of AI/ML research as the combination of pediatric and adult data and inclusion of different developmental stages of pediatric data, without training the technology on those differences, may result in algorithmic outputs that are not valid, applicable, effective or generalizable across age subgroups.

**Fig. 2:**
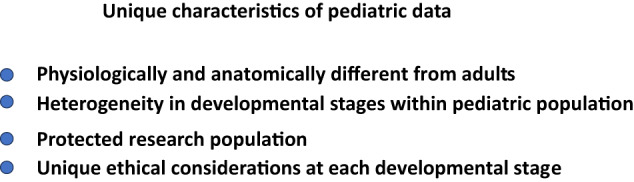
Unique characteristics of pediatric data.

Recent AI/ML studies in pediatrics have spanned a variety of body systems^19^. One study on the use of deep learning for the estimation of left ventricular ejection fraction suggests that the algorithm trained on pediatric data generalized better than an adult model when testing on pediatric cases^20^. Such findings highlight a critical need to recognize differences between pediatric and adult data in AI/ML research. While the explicit inclusion of pediatric data is important to allow for representation in age diversity across datasets, it must be done so safely and equitably (Fig. 2).

## Contents and usability of ACCEPT-AI

### Background

The generation of formalized guidelines, such as SPIRIT-AI and CONSORT-AI, have laid the foundation for safe design, conduct, reporting, and early-stage evaluation of AI/ML studies^10^. However, the lack of defined best practice standards, frameworks, or guidelines incorporating age-specific considerations for AI/ML research that involves pediatric data warrants attention. The ACCEPT-AI framework highlights fundamental ethical considerations for pediatric data use in AI/ML research. At each stage of the AI life cycle (problem selection, data collection, outcome definition, algorithm development, and post-deployment considerations), the framework promotes the evaluation and maximization of principled and ethical AI use by incorporating respect for persons, beneficence, non-maleficence, justice, transparency, and explainability in its recommendations^16^.

### Structure

ACCEPT-AI (Table 1) contains six key sections: age, communication, consent and assent, equity, protection of data, and technological considerations including transparency of techniques, training, and testing. Accompanying each section are key recommendations, which allow these ethical principles to be translated into actionable tasks by researchers, regulators, and clinicians who use or evaluate AI studies to mitigate age-related algorithmic bias.

**Table 1:** The ACCEPT-AI framework: key recommendations for pediatric data use in AI/ML research.

AGE	- Include the patient’s chronological age at the time of study enrollment - When applicable and available, include the patient’s developmental age. If unavailable, state as such - Attempt to include developmental stages and relevant milestone metrics of CYP e.g., height and weight percentile upon enrollment, to capture the heterogeneity of participants. If developmental metrics are unavailable, state them as such - Include the age(s) of intended algorithm users e.g., pediatric only, pediatric and adult, or adult only
COMMUNICATION	- Communication of study purpose to CYP as key stakeholders with developmentally appropriate communication strategies - Communication with parent(s) or legal guardians as key stakeholders - Tailor communication to social circumstance, addressing family complexities, including any court involvement - Clear communication of technology-specific study purpose, risks, benefits, and alternatives with all key stakeholders - Consider the use of videos, written material, and decision aids to facilitate education and enhance communication - Involve stakeholders, including CYP and parents, in focus groups for design feedback where possible and relevant legal and institutional permissions are obtained - State efforts taken to involve potential users in feedback of research idea and invest in community-level digital literacy - Where possible, document and articulate model explainability
CONSENT AND ASSENT	- Record mode of consent, who provided consent, e.g., parent, legal guardian, and how it was obtained - Document any complex parental relations, dynamics, or court involvement that impact consent - Document children’s social circumstances as relevant to safety, participation and evaluation - For children in state-custody, ensure consent is obtained by relevant legal guardians or custodians and documented accurately - Document relevant child protection laws pertinent to individual cases - Attain assent when developmentally appropriate and/or required by regulations - Ensure minors participate in the assent process in accordance with their developmental skills (e.g., appropriate modifications for children with clinically relevant developmental delay) - Record age when assent is provided - Ensure local laws for adolescent assent/consent are followed
EQUITY	- Ensure inclusion and exclusion criteria are clearly defined and specify disease, symptom, or condition of interest, with developmental stages considered as appropriate - State processes employed to reduce selection bias - Transparent demographic reporting, including race, documented sex, gender, and socioeconomic factors^9^ - Provide details on how gender and documented sex have been incorporated into the study design^7^ - Incorporate accessible research design to facilitate the inclusion of patients with disabilities (developmental and otherwise) - If skin tone could influence algorithmic outputs, ensure it is documented - Indicate the source of demographic information (e.g., self-reported) as well as details on non-reporting and missingness - Discuss the role of community engagement in the study
PROTECTION OF DATA	- State how data collection aligns with study objectives - State data-sharing plans when relevant - State if data is identifiable or de-identified - If data is de-identified, state compliance with the relevant legal frameworks, e.g., HIPAA, Common Rule, GDPR^24,25^ - State data protection plans, addressing unique data risks in AI/ML including protections against cybersecurity breaches. - Disclose whether data can or cannot be retrieved/removed in the future by parents and CYP - Ensure social context of child e.g., suspected or confirmed child abuse or complex social circumstance is accounted for prior to any data releases that may involve parental requests or involvement, if available
TECHNOLOGICAL CONSIDERATIONS - (TRANSPARENCY OF TECHNIQUES TRAINING, AND TESTING METHODOLOGY)	- Ensure algorithmic studies are tailored to the needs of the pediatric population and clearly documented in the study protocol - Ensure AI/ML techniques are only used when potentially beneficial to the pediatric population, and that such benefits are clearly detailed in the study protocol - Detail any potential harms that pediatric subjects may incur as a result of the study - Identify measures taken to minimize risk to pediatric subjects throughout study and post-implementation - State measures taken to monitor and document adverse events that may affect pediatric subjects - State outcome measures and plans to clinically evaluate performance of algorithms on pediatric subjects - When available, utilize validated pediatric clinical scales in the clinical algorithm evaluation - Articulate how AI/ML will be trained to recognize/account for developmental heterogeneity - Document AI/ML methods using validated guidelines, e.g CONSORT-AI and SPIRIT-AI^10,11^ - Define data input and output (e.g., images, text) as well as the source (e.g., public dataset), and output - Account for age-specific factors related to disability and developmental conditions (e.g., natural disease progression) as relevant in study design, testing, and evaluation - State if the study involves adult, pediatric, or mixed data in training and/or testing - If the study involves both adult and pediatric data, state the purpose for this combination - If the study involves both adult and pediatric data, state whether the same or separate algorithms were used to assess each group

### Vision

**Fig. 3:**
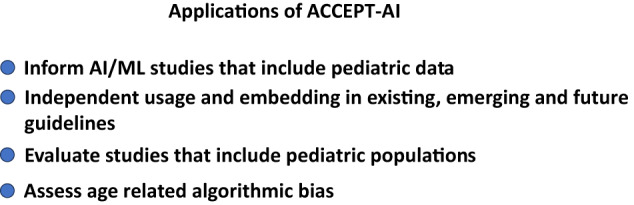
Usability of ACCEPT-AI: to be applied throughout the AI life cycle from study design to post-deployment in the above applications.

The usage of ACCEPT-AI pertains to studies designed for CYP as the primary target population for which an algorithm is applied, for adult research that incorporates pediatric data as a secondary measure, and in the utilization of public AI/ML datasets that may contain labeled or unlabeled pediatric data. While ACCEPT-AI can currently be used as an independent framework, it has also been designed to integrate into existing formalized guidelines such as CONSORT-AI and SPIRIT-AI, emerging guidelines such as TRIPOD-AI, PROBAST-AI, STARD-AI, and QUADAS-AI, and future guidelines with focus on pediatric considerations (Fig. 3)^9–13^.

### Limitations

ACCEPT-AI is a proposal that aims to provide broad guidance to meet the time-sensitive demands of rapidly emerging pediatric AI/ML studies. However, the framework has not yet undergone an E-Delphi process to strengthen expert consensus in this area, although there are plans to pursue this direction. Some sections in the ACCEPT-AI framework such as ‘Equity’ and ‘Technological considerations’ are relevant to studies across age ranges and have therefore been included for completeness despite some overlap with, for example, CONSORT-AI and SPIRIT-AI^10,11^, to allow for incorporation into current and emerging guidelines. Further, although ACCEPT-AI has been designed for a broad range of AI/ML research studies involving pediatric data, not all elements will apply to every study.

## Applications of ACCEPT-AI—illustrated examples

### Case 1: Parental consent and subject assent

*A tertiary academic center enrolls pediatric patients in a study that involves the creation of an AI/ML algorithm for assessing vascular malformations of the face. This study utilizes identifiable images of the face in its training data*.

Because CYP cannot legally consent for themselves, federal regulations include special protections for pediatric study subjects, including parental consent and assent of older pediatric subjects. It is essential that the consent process accounts for both chronological and developmental ages. Communication with the parent and subjects should include the risks, benefits, and alternatives, both at the time of enrollment and in the future, and these discussions must be documented^21^. It is important that social circumstances of the child are accounted for in the consent process, particularly if there are any sources of complexity in parent-child relationships. For children in state custody, researchers must determine if subjects may be included in the study, and if so, who is legally responsible to provide consent. Researchers must explicitly discuss relevant and important reasons to include identifiable pediatric data, such as a condition or presentation that uniquely impacts children, or where symptoms are distinct from adults. Further, whether and how subjects will be able to remove their images from a dataset in the future should be disclosed during the consent process. In instances where data has been utilized to train and test an algorithm, and cannot be removed, the likelihood that this will occur must be disclosed at the time of enrollment to key stakeholders. The ACCEPT-AI framework highlights these key considerations.

### Case 2: Communication and equity

*An AI/ML researcher plans to develop an AI algorithm for assessing pneumonia on chest X-rays in the emergency room and include adolescents. Both parental consent and participant permissions are required. The researchers wish to ensure the pediatric study population understands the risks and benefits of enrolling in this study. In addition to the study participants, they also wish to communicate their research study to the broader pediatric community to seek feedback*.

It has been acknowledged that engaging CYP in AI research is important^22^. A recent qualitative exploration of twenty-one CYP showed that they wished to contribute insights to the safe development of AI research^22^. Age-appropriate communication is the cornerstone of pediatric practice, and it is, therefore, crucial that all stakeholders are provided with relevant information on the purpose and nature of proposed AI/ML studies, and given examples of how their data may be utilized in the future. It is crucial that both chronological and developmental ages are factored into communication methods, given their relevance in several pediatric diseases.

When educating both parents and minor subjects, investigators should incorporate educational best practices. Where developmental delay is present in the subject or guardian, communication methods must be tailored appropriately. At the level of consultation with the child and family, investing in evidence-based decision aids has proven beneficial in enhancing decision-making capabilities^23^.

**Fig. 4:**
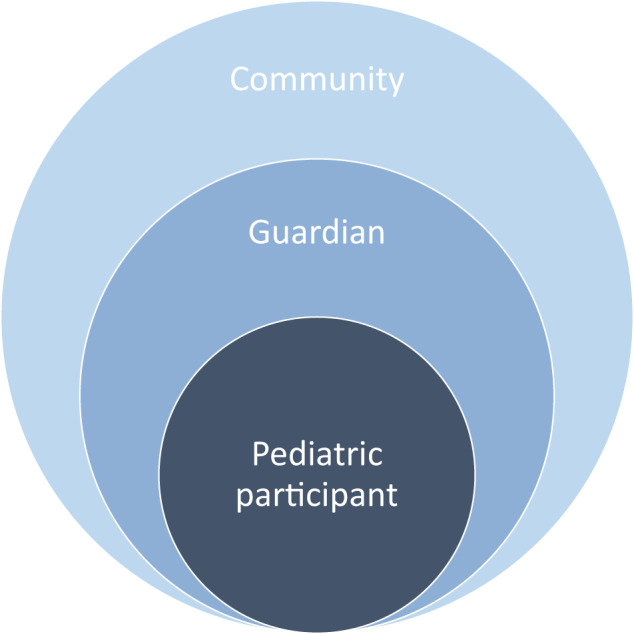
Levels of communication to improve digital literacy with key stakeholders as proposed by the ACCEPT-AI framework, adapted from McLeroy et al.^27^.

At the level of the community, efforts should be taken to improve digital literacy for young persons and parents or guardians inclusive of those from racial and ethnic minorities, rural and remote regions, and underrepresented disease groups. Collaborations with formal educational bodies to facilitate this through education on broad concepts of AI/ML health research to CYP may improve familiarity, promote transparency, clarity of research intentions, and enable exchange of ideas. Once an algorithm has been developed, further engagement in focus groups, where relevant permissions are in place, may help the iteration of working models. ACCEPT-AI emphasizes the importance of communication to improve digital literacy and engagement through the AI life cycle, at individual, parental, and community levels (Fig. 4).

### Case 3: Data protection and identification

*Researchers review a large public skin image database for the training of an ML algorithm that aims to diagnose skin lesions. They noticed unlabeled pediatric data mixed into the dataset*.

Pediatric data must only be utilized when the data and technology addresses a clear need for the pediatric population. Researchers must be transparent about needs and potential benefits for data use in their protocols, and should clearly describe measures taken to minimize risk to pediatric subjects. Adverse events should be clearly documented, with plans in the protocols for clinical evaluation using validated pediatric tools, where possible. Currently, data protection laws involving de-identifiable data In the United States do not separate adult and pediatric data. Differentiating de-identifiable and identifiable data is a key consideration for safe data regulation, as legislation surrounding consent and data protection differ for the respective categories. In the United States, HIPAA supports applying the “Safe Harbor Rule” to remove key identifiers from clinical patient data for secondary research use, or alternatively, suggests expert consensus to determine adequate de-identification for study inclusion^24^. In Europe, the General Data Protection Regulation (GDPR) stipulates the need for explicit consent, a prerequisite to data usage, and permits by the patient^25^. While the development of specific laws that are tailored to pediatric data usage may be beneficial, existing legal processes must be optimized for transparency with both pediatric subjects, their parents or legal guardians. Further, researchers must make clear in their protocols the measures taken to protect data security and be familiar with local laws for adolescent consent given their geographical variance^26^. New pediatric data collection for AI/ML should meet the highest standards for data security without compromising patient privacy, as proposed by key recommendations in ACCEPT-AI.

### Case 4: Key technological considerations for age-related algorithmic bias

*Researchers train a predictive diagnostic algorithm using chest X-rays available on a public dataset. Images contain no age labels. Both adult and pediatric X-rays are used to train the ML model. The algorithm is then applied to an adult-only population*.

**Fig. 5:**
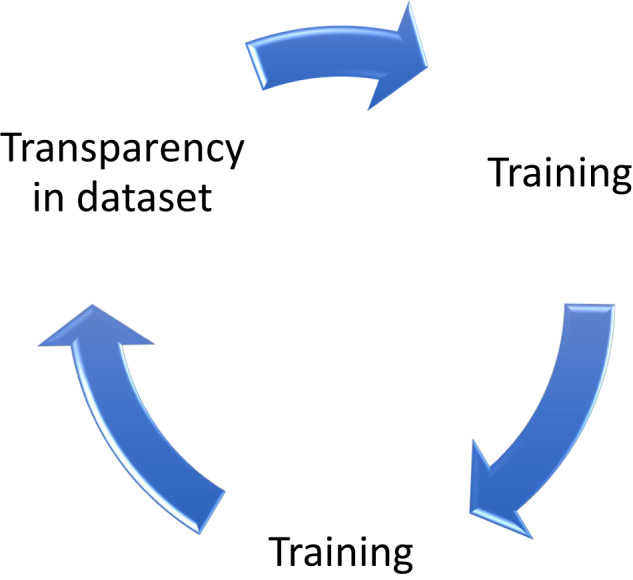
Key checkpoints for evaluating age-related algorithmic bias using the ACCEPT-AI framework.

Combining data across adults and children introduces age-related algorithmic bias, and risks compromising the applicability, generalizability, and effectiveness of a study, with potential impact on both populations. Clear documentation of the objective for which pediatric data will be collected and used in line with the ACCEPT-AI recommendations will help ensure key safety measures have been taken to avoid mixing of data unless there are clear indications to do so. Reporting the AI/ML technique applied in each study or approved device is important so that pediatric data use maps to the needs of the research question. Further, researchers should provide details on whether an algorithm has been trained to work with adult data, pediatric data, or both. While necessary at every stage of evaluation, ACCEPT-AI recommends three crucial checkpoints during an algorithmic cycle, that can be used to proactively assess for age-related bias; in dataset curation, training, and testing (including deployment and post-deployment phases) (Fig. 5).

## Conclusions

Pediatric populations face many challenges in healthcare and research settings. As the research community develops consensus guidelines for AI/ML algorithms and refines the ethical and principled use of AI, specific protections for pediatric populations are essential. Legal protections and federal mandates (e.g., enforced by the FDA) regarding the development and deployment of AI/ML algorithms for pediatric populations have also yet to be established. Here, we propose the ACCEPT-AI framework, which is a set of principles and key recommendations that can be used independently or flexibly embedded in existing, emerging, and future consensus guidelines^9–13^. The examples of ethical challenges in pediatric data utilization highlighted demonstrate the pressing need for a greater understanding of age-related bias, data source composition (e.g., combining pediatric and adult data without labeling), their analysis, and the implications for autonomy, beneficence, non-maleficence, transparency, explainability, generalizability, and fairness across pediatric and adult populations.
